# Genetic insights and therapeutic avenues: unraveling the role of polyunsaturated fatty acids as mediators between hypothyroidism and Von Willebrand disease through Mendelian randomization

**DOI:** 10.3389/fgene.2024.1426401

**Published:** 2025-01-08

**Authors:** Wenting Zhou, Rui He, Ruwei Ou

**Affiliations:** ^1^ Department of Neurology, West China Hospital, Sichuan University, Chengdu, China; ^2^ Department of Hematology, Department of Biotherapy, Cancer Center and State Key Laboratory of Biotherapy, West China Hospital, Sichuan University, Chengdu, China

**Keywords:** hypothyroidism, Von Willebrand disease, polyunsaturated fatty acids, Mendelian randomization study, mediation analysis

## Abstract

**Background:**

Previous observational studies have shown that Hypothyroidism is associated with Von Willebrand Disease (VWD), but the causal relationship has not been confirmed because of conflicting findings and confounding by mixing factors. There are also some studies suggesting that polyunsaturated fatty acids (PUFA) may be one of the potential mediators. In this study, we used a Mendelian randomization study to analyze the causal relationship between Hypothyroidism and VWD and to investigate whether polyunsaturated fatty acids mediate the effects of Hypothyroidism on VWD.

**Methods:**

Using a large publicly available genome-wide association study of predominantly European ancestry to obtain data on Hypothyroidism, VWD, and PUFA, we conducted a two-sample Mendelian randomization study to assess the causal relationship between Hypothyroidism and VWD and assess the potential role of Polyunsaturated fatty acids in mediating the causal pathway between Hypothyroidism and VWD. Finally, we also inferred reverse causality between VWD and Hypothyroidism. Inverse variance weighting (IVW) was the primary analytical method.

**Results:**

We found that Hypothyroidism may be negatively causally associated with the development of VWD and that PUFA have a role in mediating role in this process (the ratio of the mediating effect: 24.33%). The causal effects of Hypothyroidism and PUFA on VWD remained significant (p < 0.05) after correction of each other by MVMR.

**Conclusion:**

Our study unveils a novel negative correlation between hypothyroidism and VWD, further enriched by the discovery of partial mediation by PUFA. This groundbreaking finding not only advances our comprehension of VWD etiology but also opens promising avenues for its control and treatment. By elucidating the intricate interplay between hypothyroidism, PUFA, and VWD, our research pioneers a paradigm shift in therapeutic approaches, offering fresh perspectives for the management of this complex disorder.

## 1 Introduction

Von Willebrand Disease (VWD) is a common haemorrhagic disorder which includes congenital Von Willebrand Disease and acquired Von Willebrand Disease (Neff and Sidonio, 2014). Patients with VWD typically present with mucocutaneous bleeding including gum bleeding, epistaxis, easy bruising and menorrhagia (Weyand and Flood, 2021). Severe cases may lead to gastrointestinal bleeding and joint bleeding (Calmette and Clauser, 2018) and other potentially life-threatening bleeding (Pacheco et al., 2023). Studies suggest that congenital VWD has a high prevalence and incidence rate. Orphanet reports in 2022 suggest that VWD affects 0.6%–1.3% of the general population globally, with a prevalence of symptomatic VWD of approximately 10 per 100,000 people (Orphanet, 2024). And the prevalence of congential VWD is relatively consistent across different races and ethnicities (Rodeghiero et al., 1987; Werner et al., 1993). Referral prevalence estimates for VWD range from 4.4 to 16.5 per 100,000 population in Europe, from 0.1 to 8.8 per 100,000 population in the Western Pacific region, and from 1.8 to 2.0 per 100,000 population in the Eastern Mediterranean region (Du et al., 2023; Abbonizio et al., 2020; Yoon et al., 2020). Acquired VWD is a relatively rare acquired bleeding disorder that usually occurs in elderly patients (Jakway, 1992). Patients have no history of bleeding from childhood or family history of bleeding, but develop the disease as a result of a decrease in the amount or activity of the Von Willebrand factor (VWF) in plasma due to a variety of acquired factors, such as the production of autoantibodies to VWF or excessive clearance of VWF (Michiels et al., 2001). In the primary care setting, the prevalence of symptomatic VWD is at least one in 1,000 (Bowman et al., 2010). Due to assay variability, limited sensitivity of certain assays, and preanalytic issues with sample handling, diagnosis of VWD may be missed in a significant proportion of individuals with the disease. VWD has been thought to be related to a number of conditions including lymphoproliferative and myeloproliferative diseases (van Genderen and Michiels, 1998), cardiovascular diseases (Horiuchi et al., 2019), neoplasia (Nicol et al., 2022), miscellaneous disorders (Hanratty and Cowan, 2010; Scott et al., 1981), immunological disorders and drugs (Tsybul’ko et al., 2001).

Hypothyroidism is a syndrome of generalised hypometabolism due to a reduction in the synthesis and secretion of thyroid hormones or a diminished organisational effect. It is a chronic disease associated with deficiency of thyroid hormones, thyroxine (T4) and triiodothyronine (T3). The National Health and Nutrition Examination Survey (NHANES III) finds that the prevalence of Hypothyroidism in the U.S. population is 4.6% (Hollowell et al., 2002). The prevalence of hypothyroidism in different regions is different, with the prevalence of hypothyroidism in UK as high as 3%, in Japan as high as 1.16%, in Saudi Arabia as high as 5.3%, and in New Zealand as high as 2.5%, and so on (Chaker et al., 2022). Moreover, European epidemiological data estimate the prevalence of Hypothyroidism at 3.05%, with a prevalence of 5.1% in women and 0.92% in men (Garmendia Madariaga et al., 2014). The prevalence of Hypothyroidism increases with age. Cross-sectional data from the US population-based Atherosclerosis Risk in Communities (ARIC) cohort found that the prevalence of Hypothyroidism in older subjects (≥65 years) was 24% (Diab et al., 2019). It has been reported that Hypothyroidism may be associated with medications, autoimmune diseases, cardiovascular diseases and other factors (Gottwald-Hostalek and Schulte, 2022).

Several case reports and observational studies have now found a strong association between Hypothyroidism and vascular haemophilia (Bruggers et al., 1994; Coccia and Barnes, 1991; Concha et al., 2005; Dagdeviren Cakir et al., 2023). A cross-sectional study by Franchini et al. (2004) found subclinical Hypothyroidism in 8 (6.1%) of 131 individuals with low VWF levels. In a prospective study, VWD was found in 29 of 90 untreated hypothyroid patients (71 women, 19 men), with a prevalence of about 33%. In contrast, after thyroid hormone replacement therapy to restore hyperthyroidism, VWF levels were significantly elevated in almost all patients (Stuijver et al., 2014). These studies suggest that Hypothyroidism is a predisposing factor in the development of VWD. While others have suggested that Hypothyroidism is not a risk factor for VWD. Panciera and Johnson (1994) found that plasma VWF antigen concentrations in hypothyroid dogs were within reference limits and significantly reduced after levothyroxine treatment. Although these observational studies are controversial as to whether there is a positive or negative correlation between hypothyroidism and VWD, it is certain that they all agree that there is a strong relationship between hypothyroidism and VWD. What kind of relationship existed and its mechanism were not given a specific and clear explanation in these observational studies and need to be further investigated.

Research showed that Hypothyroidism may further affect lipid metabolism (Mavromati and Jornayvaz, 2021; Janota et al., 2023). Raederstorff et al. (1991) found that n-6 and n-3 polyunsaturated fatty acids (PUFA) are differently affected by the hypothyroid state through experiments in mice. In the hypothyroid state, the conversion of linoleic acid (18:2n-6) to arachidonic acid (20:4n-6) is inhibited while the conversion of linolenic acid (18:3n-3) to docosapentaenoic acid (20:5n-3) is favoured (Raederstorff et al., 1991). Epidemiological studies have shown that n-3 polyunsaturated fatty acids (PUFA) are associated with a reduced risk of atherosclerosis and hyperlipidaemia (Adkins and Kelley, 2010; Rodríguez-Cruz et al., 2005). However, current research evidence is not available on the relationship between PUFA and VWD, so we do not know if there is a potential causal relationship between the two.

There are no high-quality studies published on the relationship between Hypothyroidism, PUFA, and VWD studies, and much of the data is based solely on case reports or even non-clinical trials. In traditional observational epidemiology, the association between exposure and outcome may be affected by confounding factors and reverse causal associations, which limits its credibility in causal inference. As a result, the relationship between Hypothyroidism and VWD remains uncertain at present. Therefore, there is a need to further investigate the genetic causality between them so as to provide a theoretical basis for clinical treatment and prevention.

In recent years, there has been a gradual rise in Mendelian randomised epidemiological research methods (Davies et al., 2018). Mendelian randomization (MR) uses genetic variation as an instrumental variable to estimate the causal relationship between exposure and disease outcome (Lawlor et al., 2008). Because genotype assignment is randomised, associations between genetic variants and outcomes are not affected by common confounders. The causal sequences derived from Mendelian randomization studies are therefore plausible (Bowden and Holmes, 2019). Therefore, we hypothesized that there is a causal relationship between hypothyroidism and VWD and that PUFA mediates the relationship. We utilized two-sample Mendelian randomization (MR) analysis. We used the germline genetic variation as an instrumental variable (IV) for exposure to verify the causal relationship between VWD and Hypothyroidism and the mediating role of PUFA in it. Moreover, the mediating effect of PUFA in the risk of Hypothyroidism and VWD was further evaluated to provide new insights into the causal relationship between VWD and Hypothyroidism.

## 2 Materials and methods

### 2.1 Research design

**FIGURE 1:**
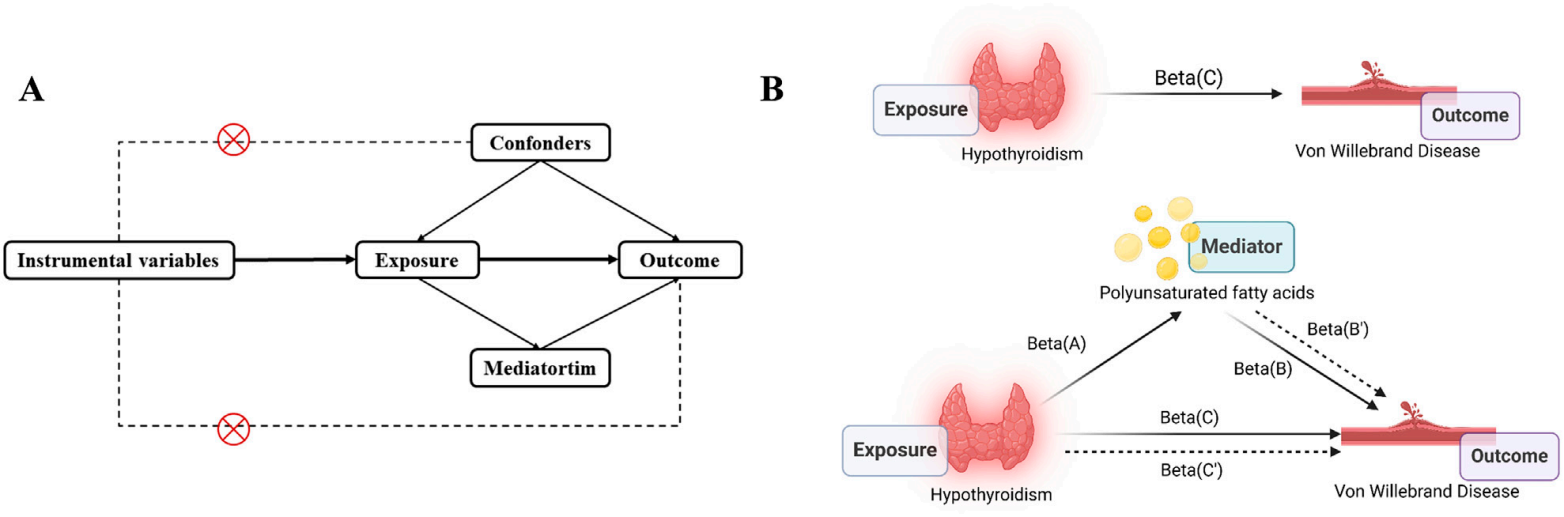
Study overview. **(A)**, The principal diagram for the Mendelian randomization study. **(B)**, Mediation analysis. Beta(A), Beta(B), Beta(C) are based on two-sample MR analyses. Beta(B’) is the effect of PUFA on VWD after correction of Hypothyroidism. Beta(C’) is the effect of Hypothyroidism on VWD after correction of PUFA. Picture B was created in BioRender. Zhou W. (2024) https://BioRender.com/w40c556.

This study used MR analysis and mediation analysis to identify and estimate the causal relationship between Hypothyroidism and VWD as well as whether this relationship can be mediated by polyunsaturated acid levels (Figure 1). The exposure variable was identified as Hypothyroidism and the outcome variable as VWD by searching for genome-wide association studies (GWAS). Firstly, we obtain the pooled data of Hypothyroidism and VWD from the GWAS database, then clarify the existence of relevant SNPs (instrumental variables), and screen out the qualified SNPs. Finally, make use of multiple statistical methods to comprehensively determine the association between Hypothyroidism and the risk of developing VWD. This study strictly followed the three hypotheses of the MR analysis: 1) the selected IVs were associated with exposure; 2) the IVs were not associated with any confounders; 3) the IVs were associated with clinical outcomes only through exposure factors (Sekula et al., 2016).

### 2.2 Data sources

Data related to the Hypothyroidism (exposure variable), VWD (Ending variable) and PUFA (mediator) were obtained from the IEU open GWAS (https://gwas.mrcieu.ac.uk/). We downloaded the GWAS summary statistics for Hypothyroidism (ID: ebi-a-GCST90018862) originating from a European population through this database, containing 379,986 controls and 30,155 cases participating, involving 24,138,872 SNPs. Data for the ending variable VWD (ID: finn-b-D3_VONVILLEBRAND) were also obtained from this database, which included European populations of both males and females, with a total of 216,099 controls and 149 cases participating, covering 16,380,461 SNPs. The mediator Polyunsaturated fatty acids (ID: met-d-PUFA) data included 114,999 sample sizes involving 12,321,875 SNPs (Supplementary Table 1).

### 2.3 Selection of IVs

Instrumental variables should satisfy the following conditions: 1) SNPs with a significance threshold (P < 5 × 10^−8^) within the locus range were used as potential instrumental variables; 2) since the presence of linkage disequilibrium (LD) can lead to bias, we set the LD of the snp that were significantly correlated with exposure to satisfy r^2^ < 0.001 and kb > 10,000 to ensure that the SNPs were independent of each other (Burgess et al., 2013); (3) SNPs with F > 10 were selected to exclude weak instrumental variables (Burgess et al., 2011). The F value was calculated as follows: f = β^2^_exposure_/SE^2^_exposure_, where β is the effect value and se is the standard error.

### 2.4 Mendelian randomization analysis

We mainly used random effects model inverse variance weighting (IVW) to determine the causal relationship between exposure and outcome, while Weighted Median (WM), MR-Egger, Simple mode and Weighted mode were used as auxiliary analyses to improve accuracy and stability (Slob and Burgess, 2020). The effect on the risk of VWD was expressed as OR and 95% CI.In MR analysis, *P* < 0.05 indicated a significant causal relationship between exposure and outcome. MVMR was performed using “Mendelian randomization” and “TwoSampleMR” R package (Sanderson, 2021). IVW was the primary analysis method and MR-Egger was the secondary analysis method.

### 2.5 Sensitivity analyses

We used MR-Egger regression to test for the presence of pleiotropy in IVs and whether it affected the results (Bowden et al., 2015). It was judged that if the MR-Egger intercept was close to 0 or *p* > 0.05, there was no effect of pleiotropy in IVs. For the IVW method, we used Cochran’s Q test to check for heterogeneity between IVs, and it was considered that results with *p* > 0.05 indicated the absence of heterogeneity (Greco M et al., 2015). The leave-one-out sensitivity test was used to analyse the effect of the remaining SNPs on the results by excluding each SNP in turn, thus observing whether the results of each analysis changed; if the change was not significant, the results were considered to be relatively robust (Zheng et al., 2017).

### 2.6 Mediated analysis

Direct effect of exposure on outcome = Beta(C’). The mediated effect was calculated as Beta = Beta(C)-Beta(C’). The proportion of the mediated effect to the total effect:R = Beta/Beta(C)*100%.

### 2.7 Data visualisation

Scatter plots are used to visualise causal effects between exposure and outcome, with the slope of the line indicating the magnitude of the causal association. Forest plots assessed genetic variance effects. Leave-one-out analyses determined whether a single SNP caused a significant change in outcome by eliminating SNPs sequentially. Publication bias was assessed by checking the symmetry of the funnel plot.

All analyses were performed using the TwoSampleMR package and R Foundation version 4.3.3. Statistical significance was set at *p* < 0.05.

## 3 Results

### 3.1 Causal effects between hypothyroidism and VWD

**FIGURE 2:**
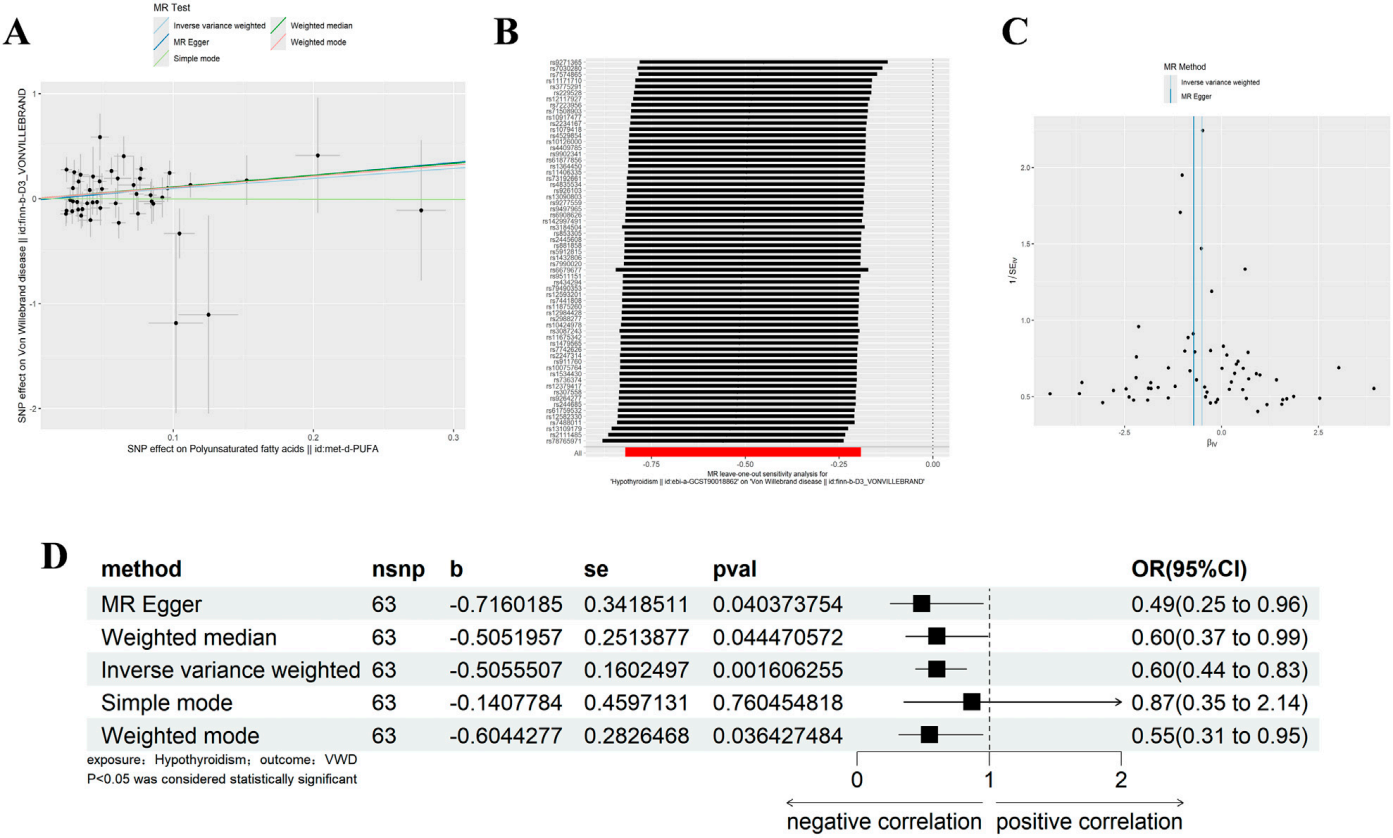
Results of different MR analysis methods for genetic correlation of Hypothyroidism and VWD. **(A)**, A scatter plot is used to visualize the causal effect between Hypothyroidism and VWD; **(B)**, Forest plot of the “leave-one-out’’ sensitivity analysis method to show the influence of individual SNPs on the results. The red point indicates the IVW estimates using all SNPs. **(C)**, A funnel plot of the relationship between the causal effect of Hypothyroidism on VWD. **(D)**, Two-sample MR analysis results for Hypothyroidism on VWD risk. VWD, von willebrand disease; nsnp, the number of single nucleotide polymorphisms; se, standard error; OR, odds ratio; CI, confidence interval.

We removed SNPs with LD in the 10,000 kb range and the most significant SNPs with r^2^ greater than 0.001, and analysed Hypothyroidism against VWD by removing palindromic sequences and SNPs with F-values of less than 10, resulting in 63 SNPs as IVs (Supplementary Table 2). The IVW results showed that Hypothyroidism was negatively correlated with VWD (β = −0.506, 95% CI:0.441–0.826, *p* = 0.002, OR = 0.603) (Figure 2D). And the β values of MR-Egger regression, WME, WM and SM were in the same direction as those of IVW. The results of Cochran’s Q-test showed no heterogeneity (*p* = 0.683) (Table 1). The analysis of the intercept term of MR-Egger regression showed that there was no horizontal multiplicity of SNPs (*p* = 0.488) (Table 2). The leave-one-out method of analysis showed that no SNPs with a large effect on the causal association estimates were found after excluding individual SNPs, demonstrating the robustness of the results. The funnel plots were symmetrical, indicating that our analyses did not affect pleiotropy (Figures 2A–C). We therefore conclude that Hypothyroidism is negatively associated with VWD.

**TABLE 1:** Sensitivity analysis of heterogeneity test.

Exposure	Outcome	MR Egger			IVW		
		Q	Q_df	Q_pval	Q	Q_df	Q_pval
Hypothyroidism	VWD	55.7329979	61	0.66657069	56.21880181	62	0.682909662
Hypothyroidism	PUFA	169.9975994	62	5.36E-12	175.5769702	63	1.45E-12
PUFA	VWD	58.20851652	45	0.089429817	58.34841074	46	0.104631709
VWD	Hypothyroidism	16.83663133	12	0.155846725	18.33155338	13	0.14533148

VWD, Von Willebrand disease; PUFA, polyunsaturated fatty acids; IVW, inverse variance weighted; Q_df, Q_ degree of freedom.

**TABLE 2:** Sensitivity analysis of horizontal pleiotropy test.

Horizontal pleiotropy test				
Exposure	Outcome	MR Egger		
		egger_intercept	se	pval
Hypothyroidism	VWD	0.024813255	0.035600265	0.488452926
Hypothyroidism	PUFA	0.003028943	0.002123362	0.158744922
PUFA	VWD	−0.016991527	0.051667748	0.743786094
VWD	Hypothyroidism	0.007358986	0.00712928	0.322324753

VWD, Von Willebrand disease; PUFA, polyunsaturated fatty acids; se, standard error; F, F-statistic.

### 3.2 Mediating role of polyunsaturated fatty acids

#### 3.2.1 Two-sample analysis of polyunsaturated fatty acids and VWD

**FIGURE 3:**
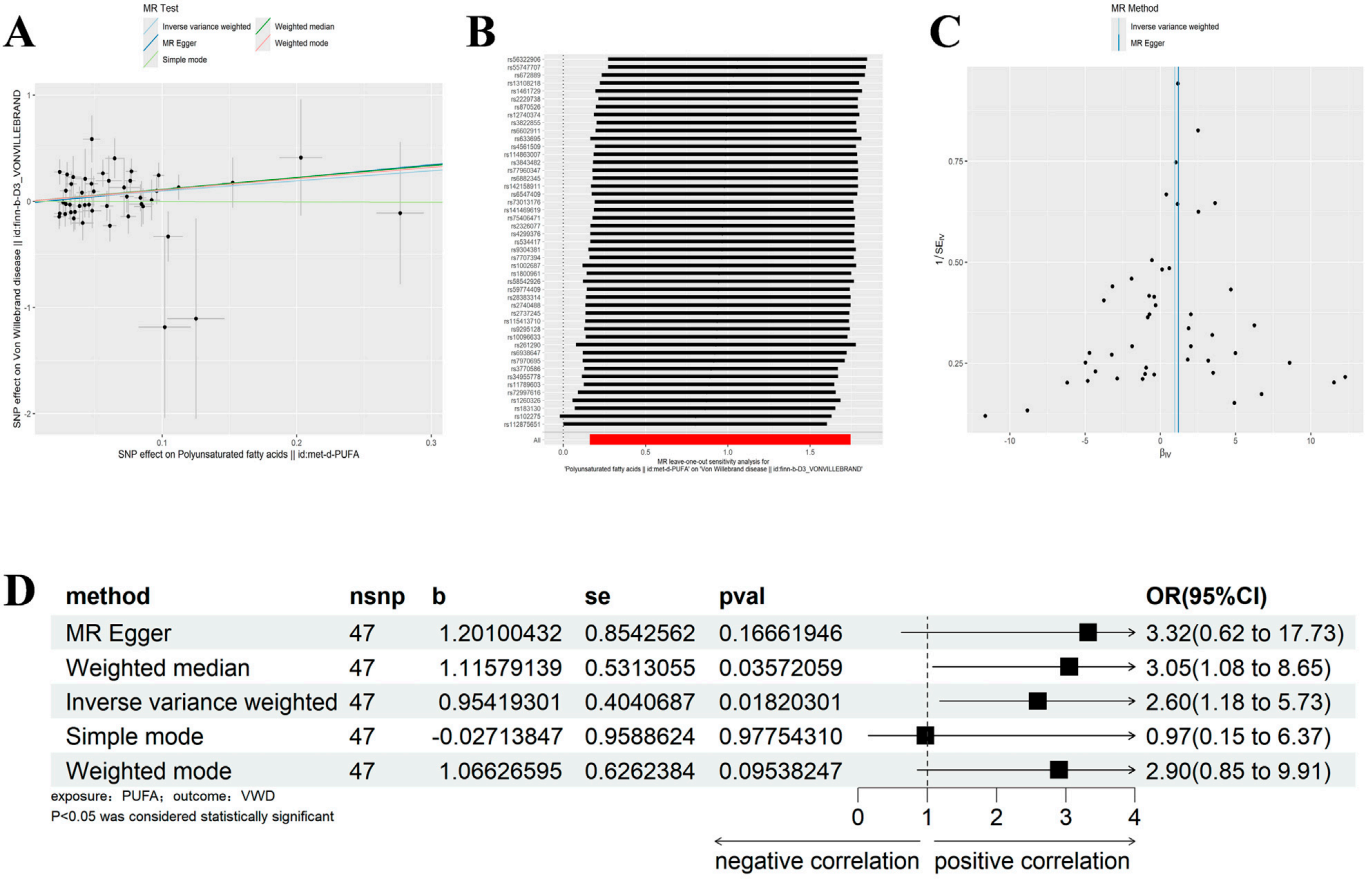
Results of different MR analysis methods for genetic correlation of PUFA and VWD. **(A)**, A scatter plot is used to visualize the causal effect between PUFA and VWD; **(B)**, Forest plot of the “leave-one-out’’ sensitivity analysis method to show the influence of individual SNPs on the results. The red point indicates the IVW estimates using all SNPs. **(C)**, A funnel plot of the relationship between the causal effect of PUFA on VWD. **(D)**, Two-sample MR analysis results for PUFA on VWD risk. PUFA, polyunsaturated fatty acids; VWD, von willebrand disease; nsnp, the number of single nucleotide polymorphisms; se, standard error; OR, odds ratio; CI, confidence interval.

Similarly, we excluded SNPs with LD by the same criteria (Kb = 10,000, r^2^ = 0.001), and analysed VWD against Polyunsaturated fatty acids to screen out the palindromic sequences, and identified 47 SNPs as IVs (Supplementary Table 2). IVW, WM results showed that Polyunsaturated fatty acids were significantly positively correlated with VWD (IVW: β = 0.954, 95%CI:1.176–5.733, *p* = 0.018, OR = 2.597; WM:β = 1.116, 95%CI:1.077–8.646, *p* = 0.036, OR = 3.052) (Figure 3D). Heterogeneity was detected using MR-Egger and IVW analyses, and no heterogeneity or horizontal pleiotropy was found in the MR analyses of VWD on Polyunsaturated fatty acids (Tables 1, 2). The leave-one-out method showed that the causality was not affected by any of the anomalous variables (Figures 3A–C). Therefore, the results suggest an increased risk of VWD with elevated Polyunsaturated fatty acids.

#### 3.2.2 Two-sample analysis of polyunsaturated fatty acids and hypothyroidism

**FIGURE 4:**
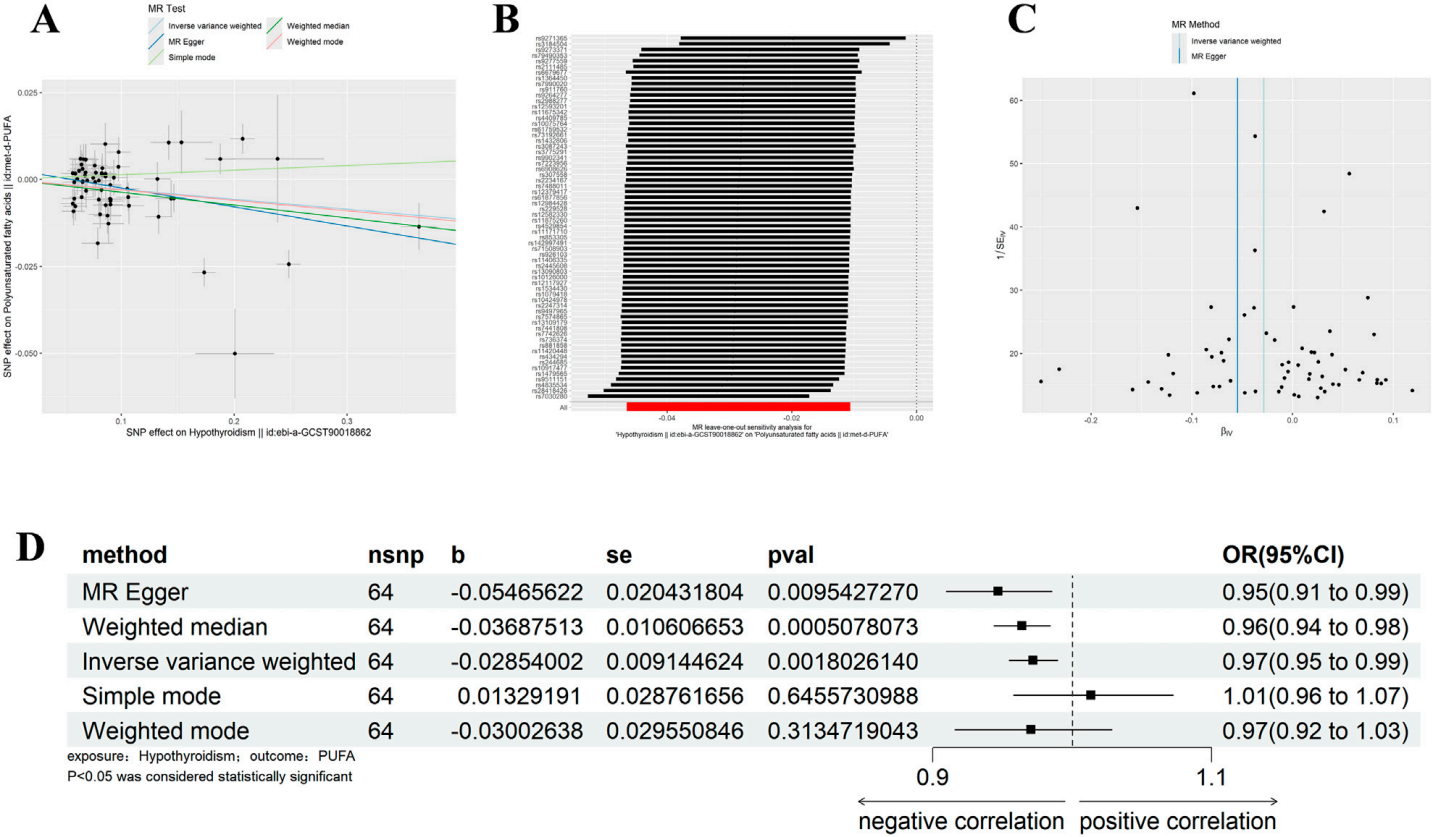
Results of different MR analysis methods for genetic correlation of Hypothyroidism and PUFA. **(A)**, A scatter plot is used to visualize the causal effect between Hypothyroidism and PUFA; **(B)**, Forest plot of the “leave-one-out” sensitivity analysis method to show the influence of individual SNPs on the results. The red point indicates the IVW estimates using all SNPs. **(C)**, A funnel plot of the relationship between the causal effect of Hypothyroidism on PUFA. **(D)**, Two-sample MR analysis results for Hypothyroidism on PUFA risk. PUFA, polyunsaturated fatty acids; SNPs, the number of single nucleotide polymorphisms; SE, standard error; OR, odds ratio; CI, confidence interval.

With the same criteria as above, we screened 65 SNPs that met the requirements (Supplementary Table 2). The results of MR Egger, WM, and IVW showed that Polyunsaturated fatty acids were significantly negatively correlated with hypothyroidism (IVW: β = −0.029, 95%CI:0.955–0.990, *p* = 0.002, OR = 0.972; MR Egger: β = −0.055, 95%CI:0.910–0.985, *p* = 0.002, OR = 0.972; MR Egger: β = −0.055, 95%CI:0.910–0.985, *p* = 0.010, OR = 0.947; WM: β = −0.037, 95%CI:0.944–0.984, *p* = 0.001, OR = 0.964) (Figure 4D). There was no horizontal pleiotropy, but heterogeneity exists, and the leave-one-out method showed robust results (Figures 4A–C) (Tables 1, 2). The results therefore suggested that hypothyroidism is negatively associated with Polyunsaturated fatty acids.

### 3.3 MVMR

We screened the IVs by MVMR analysis (Supplementary Table 3) and found that the causal effect of hypothyroidism on VWD remained significant after correction for Polyunsaturated fatty acids. Similarly, the causal effect of Polyunsaturated fatty acids on VWD was significant after correction for Hypothyroidism. We did not find horizontal pleiotropy by MR-PRESSO (*p* = 0.221) method. MVMR analyses by IVW method and MR-Egger method found no heterogeneity (IVW:test statistic for heterogeneity at 95 degrees of freedom = 107.2306, *p* = 0.1841; MR-Egger:test statistic for heterogeneity at 94 degrees of freedom = 107.1875, *p* = 0.1665) (Table 3). The results of MVMR suggest that hypothyroidism and Polyunsaturated fatty acids together influence the development of VWD.

**TABLE 3:** MVMR analysis of the association between Hypothyroidism/PUFA and VWD.

Exposure	MV-IVW				MV-egger			
	Beta	Se	95%CI	Pval	Beta	Se	95%CI	Pval
Hypothyroidism	−0.628	0.181	−0.983, −0.273	0.001	−0.668	0.274	−1.205, −0.131	0.015
VWD	0.795	0.364	0.081, 1.509	0.029	0.788	0.368	0.067, 1.509	0.032

SE, standard error; F, F-statistic; VWD, Von Willebrand disease; CI, confidence interval; MV-IVW, multivariate inverse variance weighted; MV-Egger, Multivariate MR‒Egger.

### 3.4 Mediating effect of polyunsaturated fatty acids

The total effect of hypothyroidism on VWD Beta(C) = −0.506. Corrected for Polyunsaturated fatty acids, the direct effect of hypothyroidism on VWD was Beta(B’) = -0.628. Corrected for hypothyroidism, the direct effect of Polyunsaturated fatty acids on VWD was Beta(C’) = 0.794. The mediating effect was Beta = Beta(C)-Beta(C’) = 0.123. The percentage of mediating effect in the total effect:R = Beta/Beta(C)*100% = 24.33%. The results indicated that Polyunsaturated fatty acids played a partial mediating role in the causal effect.

### 3.5 Reverse two-sample MR analysis

In the reverse two-sample analysis, we used a relaxed threshold (*p* < 5 × 10^−6^) to obtain more IVs. 14 SNPs were finally screened. IVW results showed no reverse causality between hypothyroidism and VWD when VWD was the exposure and hypothyroidism was the outcome (*p* = 0.144). Neither heterogeneity nor horizontal pleiotropy was present (Table 1).

## 4 Discussion

Through the MR analysis, we proved that the hypothesis is valid. There is a causal relationship between VWD and hypothyroidism. Unlike previous studies, there is a negative relationship between hypothyroidism and VWD. And Polyunsaturated fatty acids are shown to play a partial mediating role in this process. This may provide a new idea for the treatment of VWD, that is, it may be possible to intervene metabolic targets to achieve treatment or remission.

VWD is caused by a defect in the amount or abnormal activity of VWF and is the most common bleeding disorder (Franchini and Focosi, 2023). However, the diagnosis of VWD is complex and challenging. There is a high degree of heterogeneity among its subtypes and a lack of universally applicable diagnostic criteria. This leads to many diagnostic delays or missed diagnoses (Stonebraker et al., 2023). Joint haemorrhage occurs in up to 45% of patients with severe VWD. Recurrent joint haemorrhage can lead to severe arthropathy, affecting the patient’s daily function and compromising quality of life (van Galen et al., 2012). Recurrent gastrointestinal bleeding is also a unique symptom of VWD. This symptom is difficult to treat and is usually associated with vascular dysplasia (Weyand and Flood, 2021). Therefore, it is critical to identify risk factors for VWD. It is also necessary to study the relationship between risk factors and protective factors in order to intervene early in the treatment process.

Hypothyroidism and VWD have been associated in previous observational studies, but their results are controversial. There are more studies showing hypothyroidism as a predisposing factor for VWD, meaning that hypothyroidism is positively associated with VWD (Hanratty and Cowan, 2010; Galli-Tsinopoulou et al., 2006). In a prospective study conducted by Levesque et al. (1993), the team found a decrease in VWF synthesis during hypothyroidism. Blesing (1990) analyzed a 17-year-old hypothyroid female. Her abnormal hemostatic mechanism was similar to that exhibited by VWD. After treatment of hypothyroidism, her coagulation defects normalized (Blesing et al., 1990). Assimina Galli Tsinopoulou also considered that hypothyroidism contributed to the development of VWD in a 10-year-old girl (Galli-Tsinopoulou et al., 2008). However, the results of this study, which used Mendelian randomization, showed a significant negative association between hypothyroidism and VWD. This is in contrast to the findings of some studies. We believe that the reason for this phenomenon may be that the subjects in these observational studies are too few and of a contingent nature. It is therefore difficult to determine their representativeness. Secondly, it is because the pathogenesis of hypothyroidism and VWD is complex and affected by multiple factors, making it difficult to establish a single causal relationship. And it is impossible to avoid the influence of other confounding factors. Of course, it may also be due to the differences in research methods, sample size, and characteristics of the study population. The Mendelian randomization method used in this study circumvents these problems to a large extent. This method reduces selection bias and confounding bias that may exist in traditional observational studies and makes the results more reliable. There are also some animal experiments which also demonstrate that hypothyroidism is not a risk factor for VWD. Krogh (2014) found that exercise causes a decrease in thyroid hormone concentrations in sled dogs. However, decreased thyroid hormone concentrations were associated with increased VWF (the pathogenesis of VWD) (Krogh et al., 2014). Some scholars have suggested that levothyroxine can be an effective treatment for canine VWD. However, so far,the evidence in favor of this treatment has been lacking. Heseltine (2005) failed to determine in his experiments that levothyroxine supplementation had a direct effect on plasma VWF concentration or activity in thyroid Dobermans with VWD (Heseltine et al., 2005). Panciera and Johnson (1994) also found that plasma VWF antigen concentrations in dogs with hypothyroidism were within the reference range. However, plasma VWF antigen concentrations were surprisingly significantly reduced after treatment with thyroxine (Panciera and Johnson, 1994). Obviously, these findings are contrary to some studies, but consistent with the findings of our MR study.

Polyunsaturated fatty acids are dietary fatty acids that include n-3 polyunsaturated fatty acids and n-6 polyunsaturated fatty acids. Both have been studied for their importance to the human body. For example, n-3 polyunsaturated fatty acids may have anticonvulsant effects in humans and animals (Taha et al., 2010), and n-6 polyunsaturated fatty acids may regulate various balances, inflammatory cell activity and cytokine production within the immune system (Calder and Grimble, 2002). Many studies have demonstrated that thyroid hormones regulate lipid metabolism. Faas and Carter (1982) both found that thyroid hormones can affect the synthesis of unsaturated fatty acids through experiments in rats. Fatty acid desaturation was significantly reduced in hypothyroid rats. Valdemarsson’s study (1988) also found elevated concentrations of polyunsaturated fatty acids in hypothyroid patients following thyroxine replacement therapy. In particular, there was a positive effect on the conversion of linoleic acid to longer, more polyunsaturated fatty acids (Valdemarsson and Gustafson, 1988). These views are supported by the results of our Mendelian randomization study. Analysis of MR results showed that hypothyroidism was negatively associated with Polyunsaturated fatty acids. Treatment of hypothyroidism may favour the conversion and synthesis of polyunsaturated fatty acids. This may also provide new ideas for the treatment of some polyunsaturated fatty acid-related diseases such as epilepsy (Sinha et al., 2009) and hypercholesterolaemia (Bortz, 1974). However, there are fewer studies on the causal relationship between Polyunsaturated fatty acids and VWD. Schmidt (1992) found that after 9 months of n-3 PUFA supplementation, bleeding time increased and plasma VWF levels decreased, which is the cause of VWD (Schmidt et al., 1992). After our two-sample Mendelian randomization study, we found a significant positive correlation between the two. That is, elevated unsaturated fatty acids may be a potential risk factor for the development of VWD. We need to explore the deeper mechanism and pay attention to it.

More importantly, our study found that polyunsaturated fatty acids play a partially mediating role of 24.33% in the causal relationship between hypothyroidism and VWD. Hypothyroidism is negatively associated with VWD, meaning that treatment of Hypothyroidism may be accompanied by a risk of VWD. But this effect is partly mediated through Polyunsaturated fatty acids levels. Thus, by affecting the levels of polyunsaturated fatty acids, it is possible to reduce the risk of developing VWD as a result of treating hypothyroidism. Polyunsaturated fatty acids have the potential to be a new metabolic target for the future treatment or mitigation of the development of VWD. This needs to be further explored and investigated in the future.

**FIGURE 5:**
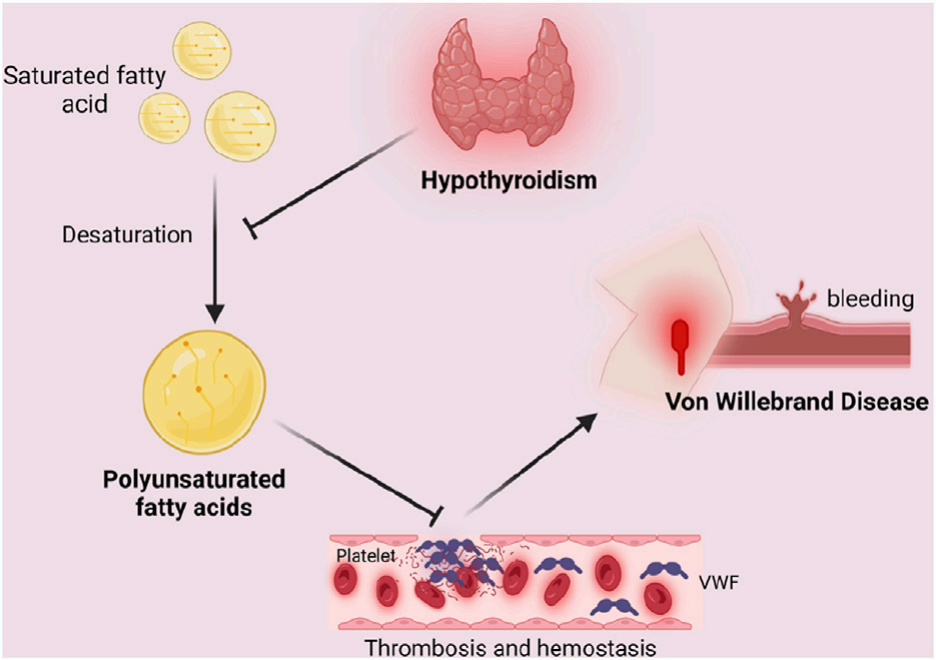
Mediating role of polyunsaturated fatty acids in the causal relationship between hypothyroidism and Von Willebrand Disease. Unsaturated fatty acids lead to a decrease in the number of VWF, which results in endothelial repair malfunction and increases bleeding time, leading to coagulation dysfunction and inducing the development of VWD. Hypothyroidism inhibits the desaturation of saturated fatty acids, thus blocking the above process by inhibiting the synthesis of polyunsaturated fatty acids, resulting in the alleviation of the symptoms or development of VWD. Created in BioRender. Zhou W. (2024) https://BioRender.com/u98s606.

Based on our results, PUFA play a mediating role in the relationship between hypothyroidism and VWD. So how does hypothyroidism affect the development of VWD by mediating PUFA? It is well known that thyroid hormones are involved in lipid metabolism. Studies have shown that thyroid hormones can influence the activity of the enzyme desaturase, which is involved in the synthesis of PUFA. Hypothyroidism leads to a decrease in delta six desaturase activity, which results in a decrease in delta six unsaturated fatty acids (linoleate) (Faas and Carter, 1982). Raederstorff (1991) demonstrated in rat experiments that hypothyroidism can affect the competition between n-3 and n-6 PUFA for desaturase, elongase, and acyltransferase. Impaired activities of elongase and desaturase in hypothyroid rats ultimately affect PUFA synthesis (Raederstorff et al., 1991). PUFA have been reported to have an effect on the synthesis of coagulation factor VWF. In PUFA-enriched endothelial cells, the number of mRNA copies of miR-29a-3p targets (i.e., coagulation factors PAI-1, TF, and vWF, as well as pro-inflammatory cytokines IL-1β, IL-6, and IL-8) was reduced (Maucher et al., 2020). In a clinical trial by Seljeflot (1998), VWF was found to be significantly reduced by PUFA supplementation (Seljeflot et al., 1998). This suggests that PUFA can lead to a decrease in the number of VWF and play a role in the development of VWD. Therefore, we believe that hypothyroidism alleviates the development of VWD by inhibiting the synthesis of PUFA (Figure 5).

This study is the first to use genetic variation as an instrumental variable to infer a causal association between Hypothyroidism and VWD, reducing the interference of confounding factors in observational studies. Hypothyroidism was found to be significantly negatively associated with VWD by our MR analysis. We chose genetic variants as exposure factors to make the findings more reliable. We also used the most recent data from the GWAS of European ancestry, which can effectively avoid the interference of confounding factors and improve the validity of the test.

However, there are some limitations. Firstly, the GWAS dataset included in the MR analyses was from Europe, and further studies on populations in other countries are needed to improve the generalisability of the results. In addition, despite the efforts to control for confounding factors in the Mendelian randomization analysis of this study, there may still be some unconsidered factors that influence the results of the study. For example, some unpublished SNPs may be associated with polyunsaturated fatty acids and VWD. This may have affected our study. In addition, we studied mainly acquired VWD, but the VWD GWAS data used did not distinguish between congenital and acquired VWD. The current GWAS data on acquired VWD has yet to be updated and improved. Additionally, the GWAS data used in the MR study came from participants of European origin. But the participants selected for the observational study were from different regions. Therefore the results may be somewhat biased. Considering these limitations, future studies could start by verifying causality and exploring potential mechanisms in order to inform relevant clinical guidelines and recommendations.

## 5 Conclusion

In conclusion, our study unveils compelling genetic evidence linking hypothyroidism to VWD through MR analysis, illuminating a previously unrecognized negative correlation. Furthermore, our findings suggest that Polyunsaturated fatty acids exert a partial mediating effect in this relationship. These insights not only deepen our understanding of VWD pathogenesis but also unveil novel avenues for therapeutic interventions. By shedding light on the intricate mechanisms underlying VWD development, our research paves the way for targeted treatment strategies aimed at ameliorating this debilitating condition.

## Data Availability

The original contributions presented in the study are included in the article/Supplementary Material, further inquiries can be directed to the corresponding authors.
