# Supplementary material for: Genetic insights and therapeutic avenues: unraveling the role of polyunsaturated fatty acids as mediators between hypothyroidism and Von Willebrand disease through Mendelian randomization
Source: Front Genet. 2025 Jan 8;15:1426401. doi: 10.3389/fgene.2024.1426401 (PMC11750860; doi:10.3389/fgene.2024.1426401)
Supplement: Supplementary file 1 [file Table1.docx]

**1.Supplementary Table 1**

Brief description of the genome-wide association study data used in this study.

| **Phenotype** | **GWAS ID** | **Sample Size** | **N case** | **N control** | **Population** | **N SNPs** |  |
| --- | --- | --- | --- | --- | --- | --- | --- |
| **Hypothyroidism** | ebi-a-GCST90018862 | 410,141 | 30,155 | 379,986 | European | 24,138,872 |  |
| **Von Willebrand Disease** | finn-b-D3_VONVILLEBRAND | 216,248 | 149 | 216,099 | European | 16,380,461 |  |
| **Polyunsaturated Fatty Acids** | met-d-PUFA | 114,999 | - | - | European | 12,321,875 |  |

**2.Supplementary Table 2**

2.1Information on instrumental variables of Hypothyroidism and VWD

| **SNP** | **Chr** | **EA** | **OA** | **Exposure** | | | **Outcome** | | | **F** |
| --- | --- | --- | --- | --- | --- | --- | --- | --- | --- | --- |
|  |  |  |  | **Beta** | **SE** | **Pval** | **Beta** | **SE** | **Pval** |  |
| rs10075764 | 5 | G | A | -0.057 | 0.0104 | 4.26E-08 | -0.09 | 0.1187 | 0.4485 | 30.03868488 |
| rs10126000 | 9 | A | C | -0.0683 | 0.0104 | 5.13E-11 | 0.1287 | 0.1229 | 0.295 | 43.12932001 |
| rs10424978 | 19 | A | C | -0.0775 | 0.0102 | 2.77E-14 | -0.0262 | 0.1188 | 0.8253 | 57.72991841 |
| rs1079418 | 6 | G | A | -0.0657 | 0.011 | 2.14E-09 | 0.1567 | 0.1319 | 0.2346 | 35.67329712 |
| rs10917477 | 1 | G | A | 0.064 | 0.01 | 1.75E-10 | -0.1574 | 0.1162 | 0.1758 | 40.95980026 |
| rs11171710 | 12 | A | G | -0.0698 | 0.01 | 3.19E-12 | 0.2513 | 0.1178 | 0.0328398 | 48.72016242 |
| rs11406335 | 13 | TG | T | -0.057 | 0.0103 | 3.44E-08 | 0.1085 | 0.1194 | 0.3633 | 30.62479175 |
| rs11675342 | 2 | T | C | 0.0906 | 0.01 | 1.40E-19 | 0.0122 | 0.1173 | 0.9175 | 82.08319973 |
| rs11875260 | 18 | G | A | 0.0751 | 0.0135 | 2.54E-08 | 0.0482 | 0.1541 | 0.7543 | 30.94640602 |
| rs12117927 | 1 | A | C | 0.0627 | 0.0105 | 2.29E-09 | -0.2298 | 0.1207 | 0.0569902 | 35.6577853 |
| rs12379417 | 9 | A | G | 0.0583 | 0.0103 | 1.51E-08 | 0.0981 | 0.12 | 0.4137 | 32.03764187 |
| rs12582330 | 12 | T | G | -0.061 | 0.0109 | 2.05E-08 | -0.1545 | 0.1247 | 0.2154 | 31.31875983 |
| rs12593201 | 15 | A | G | 0.0905 | 0.0112 | 7.69E-16 | 0.0012 | 0.1322 | 0.9929 | 65.29185317 |
| rs12984428 | 19 | A | G | -0.0659 | 0.0102 | 1.11E-10 | -0.0361 | 0.1207 | 0.7652 | 41.7415304 |
| rs13090803 | 3 | T | G | 0.0829 | 0.0128 | 9.00E-11 | -0.1138 | 0.1689 | 0.5006 | 41.94565727 |
| rs13109179 | 4 | A | G | 0.0647 | 0.01 | 9.42E-11 | 0.2543 | 0.1169 | 0.02954 | 41.86069587 |
| rs1364450 | 6 | C | A | 0.0886 | 0.0139 | 1.97E-10 | -0.1449 | 0.1584 | 0.3601 | 40.62896186 |
| rs142997491 | 16 | G | A | 0.2385 | 0.0412 | 7.02E-09 | -0.1533 | 0.3911 | 0.6951 | 33.51044669 |
| rs1432806 | 5 | G | A | 0.0583 | 0.0105 | 2.89E-08 | -0.0084 | 0.126 | 0.9466 | 30.82878391 |
| rs1479565 | 5 | A | G | 0.0975 | 0.0101 | 7.53E-22 | 0.0047 | 0.1175 | 0.968 | 93.18893877 |
| rs1534430 | 2 | T | C | -0.086 | 0.0101 | 1.44E-17 | -0.0367 | 0.1177 | 0.7554 | 72.50234226 |
| rs2111485 | 2 | G | A | 0.0813 | 0.0102 | 1.43E-15 | 0.2459 | 0.1179 | 0.03697 | 63.52996702 |
| rs2234167 | 1 | A | G | 0.0825 | 0.015 | 3.75E-08 | -0.2529 | 0.1793 | 0.1585 | 30.24985249 |
| rs2247314 | 6 | C | T | -0.086 | 0.0104 | 1.06E-16 | -0.0329 | 0.1208 | 0.785301 | 68.37984407 |
| rs229528 | 22 | T | C | 0.0903 | 0.01 | 2.31E-19 | -0.1981 | 0.1187 | 0.0951196 | 81.54050238 |
| rs2445608 | 8 | A | G | -0.0593 | 0.0101 | 3.79E-09 | 0.0243 | 0.1189 | 0.8383 | 34.47184445 |
| rs244685 | 5 | G | T | -0.0858 | 0.0132 | 7.06E-11 | -0.0851 | 0.1338 | 0.525 | 42.24979397 |
| rs2988277 | 1 | T | C | 0.0593 | 0.0106 | 2.49E-08 | 0.0695 | 0.1325 | 0.600101 | 31.2964832 |
| rs307558 | 3 | A | G | -0.0688 | 0.0119 | 8.01E-09 | -0.1279 | 0.1372 | 0.3511 | 33.42572501 |
| rs3087243 | 2 | A | G | -0.1466 | 0.0102 | 4.77E-47 | 0.0371 | 0.1232 | 0.7636 | 206.569158 |
| rs3184504 | 12 | C | T | -0.1734 | 0.0102 | 7.50E-65 | 0.0899 | 0.118 | 0.4459 | 288.9985907 |
| rs3775291 | 4 | T | C | -0.0649 | 0.0108 | 1.65E-09 | 0.2869 | 0.1253 | 0.02201 | 36.11102075 |
| rs434294 | 5 | G | A | -0.0683 | 0.0109 | 3.36E-10 | -0.0137 | 0.1247 | 0.9125 | 39.26325437 |
| rs4409785 | 11 | C | T | 0.1069 | 0.0133 | 8.04E-16 | -0.1463 | 0.1554 | 0.3464 | 64.60260204 |
| rs4529854 | 10 | T | C | -0.0768 | 0.0107 | 6.56E-13 | 0.1401 | 0.1298 | 0.2804 | 51.51726123 |
| rs4835534 | 4 | C | T | -0.1421 | 0.0132 | 7.06E-27 | 0.1347 | 0.1779 | 0.4491 | 115.887922 |
| rs5912815 | X | G | T | -0.0511 | 0.0084 | 1.05E-09 | 0.0147 | 0.1115 | 0.8949 | 37.00676398 |
| rs61759532 | 17 | T | C | 0.0905 | 0.0122 | 1.42E-13 | 0.1283 | 0.1483 | 0.3868 | 55.02694209 |
| rs61877856 | 11 | T | C | -0.0658 | 0.0115 | 1.14E-08 | 0.1491 | 0.1381 | 0.2804 | 32.73813903 |
| rs6679677 | 1 | A | C | 0.3637 | 0.0159 | 2.39E-115 | -0.1768 | 0.1622 | 0.2757 | 523.2271072 |
| rs6908626 | 6 | T | G | 0.1441 | 0.0141 | 2.04E-24 | -0.0992 | 0.1817 | 0.584901 | 104.4449914 |
| rs7030280 | 9 | T | C | 0.2075 | 0.0108 | 1.02E-82 | -0.2203 | 0.1216 | 0.0700697 | 369.1361458 |
| rs71508903 | 10 | T | C | 0.0934 | 0.0125 | 9.34E-14 | -0.2058 | 0.1497 | 0.1693 | 55.83051175 |
| rs7223956 | 17 | C | T | -0.0902 | 0.0144 | 4.27E-10 | 0.2515 | 0.167 | 0.1322 | 39.23611268 |
| rs73192661 | 3 | T | C | -0.1061 | 0.01 | 4.05E-26 | 0.0916 | 0.1196 | 0.4435 | 112.5715511 |
| rs736374 | 11 | A | G | 0.0832 | 0.0103 | 6.00E-16 | 0.0465 | 0.1214 | 0.701501 | 65.24843288 |
| rs7441808 | 4 | G | A | 0.0766 | 0.0111 | 5.17E-12 | 0.0193 | 0.1286 | 0.8804 | 47.62220102 |
| rs7488011 | 12 | T | C | 0.1052 | 0.0111 | 2.52E-21 | 0.0719 | 0.1333 | 0.5897 | 89.82214133 |
| rs7574865 | 2 | G | T | -0.1321 | 0.0117 | 1.67E-29 | 0.2824 | 0.1378 | 0.0403302 | 127.4769881 |
| rs7742626 | 6 | C | T | 0.0686 | 0.0116 | 3.41E-09 | 0.1066 | 0.1527 | 0.485 | 34.97277833 |
| rs78765971 | 1 | G | GAC | 0.2444 | 0.0162 | 1.68E-51 | 0.1481 | 0.1831 | 0.4185 | 227.5989511 |
| rs79490353 | 13 | C | T | 0.2006 | 0.0349 | 8.82E-09 | 0.1876 | 0.4981 | 0.7065 | 33.03763005 |
| rs7990020 | 13 | C | A | 0.0577 | 0.0101 | 9.97E-09 | -0.0058 | 0.1199 | 0.9616 | 32.63673919 |
| rs853305 | 8 | C | T | -0.0802 | 0.0111 | 4.40E-13 | 0.0349 | 0.1425 | 0.8065 | 52.20362499 |
| rs881858 | 6 | A | G | 0.0665 | 0.0108 | 8.46E-10 | -0.0248 | 0.1256 | 0.8434 | 37.9134811 |
| rs911760 | 9 | A | C | 0.0879 | 0.0125 | 1.95E-12 | 0.0617 | 0.1425 | 0.6651 | 49.44878287 |
| rs926103 | 1 | C | T | -0.0678 | 0.0104 | 7.65E-11 | 0.0811 | 0.1195 | 0.4974 | 42.50016257 |
| rs9264277 | 6 | C | T | -0.0862 | 0.0111 | 9.03E-15 | -0.0773 | 0.1325 | 0.56 | 60.30682385 |
| rs9271365 | 6 | G | T | 0.2484 | 0.0105 | 4.91E-123 | -0.2517 | 0.1274 | 0.0480895 | 559.6576791 |
| rs9277559 | 6 | C | T | -0.133 | 0.012 | 1.86E-28 | 0.097 | 0.1459 | 0.5063 | 122.8396788 |
| rs9497965 | 6 | T | C | 0.0827 | 0.0102 | 3.71E-16 | -0.0674 | 0.1237 | 0.586001 | 65.73679978 |
| rs9511151 | 13 | A | G | -0.0976 | 0.0106 | 3.08E-20 | 0.0276 | 0.1217 | 0.8206 | 84.77851147 |
| rs9902341 | 17 | T | C | 0.0801 | 0.0129 | 4.68E-10 | -0.1455 | 0.1447 | 0.3145 | 38.55524736 |

2.2Information on instrumental variables of Hypothyroidism and PUFA

| **SNP** | **Chr** | **EA** | **OA** | **Exposure** | | | **Outcome** | | | **F** |
| --- | --- | --- | --- | --- | --- | --- | --- | --- | --- | --- |
|  |  |  |  | **Beta** | **SE** | **Pval** | **Beta** | **SE** | **Pval** |  |
| rs10075764 | 5 | G | A | -0.057 | 0.0104 | 4.26E-08 | 0.00695811 | 0.00426399 | 0.11 | 30.03868488 |
| rs10126000 | 9 | A | C | -0.0683 | 0.0104 | 5.13E-11 | -0.00201711 | 0.00418259 | 0.75 | 43.12932001 |
| rs10424978 | 19 | A | C | -0.0775 | 0.0102 | 2.77E-14 | -0.00198617 | 0.00415399 | 0.48 | 57.72991841 |
| rs1079418 | 6 | G | A | -0.0657 | 0.011 | 2.14E-09 | -0.00301504 | 0.00437443 | 0.4 | 35.67329712 |
| rs10917477 | 1 | G | A | 0.064 | 0.01 | 1.75E-10 | 0.00592764 | 0.00405311 | 0.12 | 40.95980026 |
| rs11171710 | 12 | A | G | -0.0698 | 0.01 | 3.19E-12 | 0.000309704 | 0.00407856 | 0.97 | 48.72016242 |
| rs11406335 | 13 | TG | T | -0.057 | 0.0103 | 3.44E-08 | 0.00162102 | 0.00408904 | 0.69 | 30.62479175 |
| rs11406335 | 13 | TG | T | -0.057 | 0.0103 | 3.44E-08 | -0.0018246 | 0.00408744 | 0.649999 | 30.62479175 |
| rs11420448 | 2 | GT | G | 0.1533 | 0.0275 | 2.39E-08 | 0.0106784 | 0.00905155 | 0.2 | 31.07540549 |
| rs11675342 | 2 | T | C | 0.0906 | 0.01 | 1.40E-19 | -0.00572105 | 0.00407795 | 0.15 | 82.08319973 |
| rs11875260 | 18 | G | A | 0.0751 | 0.0135 | 2.54E-08 | 0.000465877 | 0.0057013 | 0.98 | 30.94640602 |
| rs12117927 | 1 | A | C | 0.0627 | 0.0105 | 2.29E-09 | 0.00251129 | 0.00414607 | 0.55 | 35.6577853 |
| rs12379417 | 9 | A | G | 0.0583 | 0.0103 | 1.51E-08 | -0.000798538 | 0.00418443 | 0.93 | 32.03764187 |
| rs12582330 | 12 | T | G | -0.061 | 0.0109 | 2.05E-08 | -0.000105878 | 0.00454087 | 0.84 | 31.31875983 |
| rs12593201 | 15 | A | G | 0.0905 | 0.0112 | 7.69E-16 | -0.00724742 | 0.00465493 | 0.0759994 | 65.29185317 |
| rs12984428 | 19 | A | G | -0.0659 | 0.0102 | 1.11E-10 | 0.000521514 | 0.0040954 | 0.67 | 41.7415304 |
| rs13090803 | 3 | T | G | 0.0829 | 0.0128 | 9.00E-11 | 0.00142141 | 0.00495501 | 0.86 | 41.94565727 |
| rs13109179 | 4 | A | G | 0.0647 | 0.01 | 9.42E-11 | 0.00428597 | 0.00409943 | 0.33 | 41.86069587 |
| rs1364450 | 6 | C | A | 0.0886 | 0.0139 | 1.97E-10 | -0.0126977 | 0.00573357 | 0.0290001 | 40.62896186 |
| rs142997491 | 16 | G | A | 0.2385 | 0.0412 | 7.02E-09 | 0.00593661 | 0.0183469 | 0.74 | 33.51044669 |
| rs1432806 | 5 | G | A | 0.0583 | 0.0105 | 2.89E-08 | -0.00551649 | 0.00424162 | 0.14 | 30.82878391 |
| rs1479565 | 5 | A | G | 0.0975 | 0.0101 | 7.53E-22 | 0.00361985 | 0.00414937 | 0.58 | 93.18893877 |
| rs1534430 | 2 | T | C | -0.086 | 0.0101 | 1.44E-17 | -0.000811021 | 0.0041356 | 0.98 | 72.50234226 |
| rs2111485 | 2 | G | A | 0.0813 | 0.0102 | 1.43E-15 | -0.0100176 | 0.00411205 | 0.0109999 | 63.52996702 |
| rs2234167 | 1 | A | G | 0.0825 | 0.015 | 3.75E-08 | -0.00303758 | 0.00590488 | 0.52 | 30.24985249 |
| rs2247314 | 6 | C | T | -0.086 | 0.0104 | 1.06E-16 | -0.00161344 | 0.00425956 | 0.760001 | 68.37984407 |
| rs229528 | 22 | T | C | 0.0903 | 0.01 | 2.31E-19 | -0.00159298 | 0.00408803 | 0.85 | 81.54050238 |
| rs2445608 | 8 | A | G | -0.0593 | 0.0101 | 3.79E-09 | -0.00166804 | 0.00409563 | 0.77 | 34.47184445 |
| rs244685 | 5 | G | T | -0.0858 | 0.0132 | 7.06E-11 | -0.0102009 | 0.0060841 | 0.0619998 | 42.24979397 |
| rs28418426 | 6 | C | T | 0.1877 | 0.0133 | 2.21E-45 | 0.00587893 | 0.00442464 | 0.17 | 199.1696433 |
| rs2988277 | 1 | T | C | 0.0593 | 0.0106 | 2.49E-08 | -0.0077087 | 0.00412213 | 0.0810009 | 31.2964832 |
| rs307558 | 3 | A | G | -0.0688 | 0.0119 | 8.01E-09 | 0.00326852 | 0.00500959 | 0.64 | 33.42572501 |
| rs3087243 | 2 | A | G | -0.1466 | 0.0102 | 4.77E-47 | 0.00546309 | 0.00403911 | 0.0649995 | 206.569158 |
| rs3184504 | 12 | C | T | -0.1734 | 0.0102 | 7.50E-65 | 0.0267131 | 0.00403671 | 1.30E-11 | 288.9985907 |
| rs3775291 | 4 | T | C | -0.0649 | 0.0108 | 1.65E-09 | 0.00510647 | 0.00440511 | 0.23 | 36.11102075 |
| rs434294 | 5 | G | A | -0.0683 | 0.0109 | 3.36E-10 | -0.00574717 | 0.00431702 | 0.12 | 39.26325437 |
| rs4409785 | 11 | C | T | 0.1069 | 0.0133 | 8.04E-16 | -0.00754734 | 0.00531918 | 0.0980009 | 64.60260204 |
| rs4529854 | 10 | T | C | -0.0768 | 0.0107 | 6.56E-13 | 0.000797007 | 0.00422097 | 0.69 | 51.51726123 |
| rs4835534 | 4 | C | T | -0.1421 | 0.0132 | 7.06E-27 | -0.0106287 | 0.00493502 | 0.0460002 | 115.887922 |
| rs61759532 | 17 | T | C | 0.0905 | 0.0122 | 1.42E-13 | -0.00620282 | 0.00481279 | 0.3 | 55.02694209 |
| rs61877856 | 11 | T | C | -0.0658 | 0.0115 | 1.14E-08 | 0.000714657 | 0.00448586 | 0.91 | 32.73813903 |
| rs6679677 | 1 | A | C | 0.3637 | 0.0159 | 2.39E-115 | -0.0135286 | 0.00669344 | 0.0659994 | 523.2271072 |
| rs6908626 | 6 | T | G | 0.1441 | 0.0141 | 2.04E-24 | -0.00551802 | 0.00530039 | 0.32 | 104.4449914 |
| rs7030280 | 9 | T | C | 0.2075 | 0.0108 | 1.02E-82 | 0.011697 | 0.00428808 | 0.00409996 | 369.1361458 |
| rs71508903 | 10 | T | C | 0.0934 | 0.0125 | 9.34E-14 | 0.000527308 | 0.00515007 | 0.87 | 55.83051175 |
| rs7223956 | 17 | C | T | -0.0902 | 0.0144 | 4.27E-10 | 0.00555555 | 0.00576677 | 0.27 | 39.23611268 |
| rs73192661 | 3 | T | C | -0.1061 | 0.01 | 4.05E-26 | 0.00507135 | 0.00407147 | 0.13 | 112.5715511 |
| rs736374 | 11 | A | G | 0.0832 | 0.0103 | 6.00E-16 | 0.00326369 | 0.00420343 | 0.630001 | 65.24843288 |
| rs7441808 | 4 | G | A | 0.0766 | 0.0111 | 5.17E-12 | 0.00402573 | 0.00439919 | 0.3 | 47.62220102 |
| rs7488011 | 12 | T | C | 0.1052 | 0.0111 | 2.52E-21 | -0.00274636 | 0.00453696 | 0.35 | 89.82214133 |
| rs7574865 | 2 | G | T | -0.1321 | 0.0117 | 1.67E-29 | -0.000127158 | 0.00482959 | 0.92 | 127.4769881 |
| rs7742626 | 6 | C | T | 0.0686 | 0.0116 | 3.41E-09 | 0.00574118 | 0.00448744 | 0.13 | 34.97277833 |
| rs79490353 | 13 | C | T | 0.2006 | 0.0349 | 8.82E-09 | -0.0500964 | 0.0128967 | 0.000179999 | 33.03763005 |
| rs7990020 | 13 | C | A | 0.0577 | 0.0101 | 9.97E-09 | -0.00916363 | 0.00404932 | 0.0539995 | 32.63673919 |
| rs853305 | 8 | C | T | -0.0802 | 0.0111 | 4.40E-13 | 0.000310343 | 0.00431401 | 0.86 | 52.20362499 |
| rs881858 | 6 | A | G | 0.0665 | 0.0108 | 8.46E-10 | 0.00585307 | 0.0043651 | 0.12 | 37.9134811 |
| rs911760 | 9 | A | C | 0.0879 | 0.0125 | 1.95E-12 | -0.0104188 | 0.00522985 | 0.0350002 | 49.44878287 |
| rs926103 | 1 | C | T | -0.0678 | 0.0104 | 7.65E-11 | -0.00110445 | 0.00425919 | 0.85 | 42.50016257 |
| rs9264277 | 6 | C | T | -0.0862 | 0.0111 | 9.03E-15 | 0.00737927 | 0.00419036 | 0.8 | 60.30682385 |
| rs9271365 | 6 | G | T | 0.2484 | 0.0105 | 4.91E-123 | -0.0243384 | 0.00406417 | 8.70E-07 | 559.6576791 |
| rs9273371 | 6 | T | C | 0.0791 | 0.014 | 1.65E-08 | -0.0183265 | 0.00452458 | 7.00E-05 | 31.92234433 |
| rs9277559 | 6 | C | T | -0.133 | 0.012 | 1.86E-28 | 0.0107444 | 0.00487183 | 0.021 | 122.8396788 |
| rs9497965 | 6 | T | C | 0.0827 | 0.0102 | 3.71E-16 | 0.00179297 | 0.00410938 | 0.760001 | 65.73679978 |
| rs9511151 | 13 | A | G | -0.0976 | 0.0106 | 3.08E-20 | -0.00790341 | 0.00424895 | 0.0580003 | 84.77851147 |
| rs9902341 | 17 | T | C | 0.0801 | 0.0129 | 4.68E-10 | -0.00581001 | 0.00542942 | 0.39 | 38.55524736 |

2.3Information on instrumental variables of VWD and PUFA

| **SNP** | **Chr** | **EA** | **OA** | **Exposure** | | | **Outcome** | | |
| --- | --- | --- | --- | --- | --- | --- | --- | --- | --- |
|  |  |  |  | **Beta** | **SE** | **Pval** | **Beta** | **SE** | **Pval** |
| rs1002687 | 1 | A | G | 0.0959946 | 0.00420718 | 2.00E-120 | 0.0983 | 0.1285 | 0.4441 |
| rs10096633 | 8 | T | C | -0.0339303 | 0.00610477 | 3.10E-08 | -0.2282 | 0.196 | 0.2444 |
| rs102275 | 11 | C | T | -0.0974089 | 0.00421144 | 3.20E-125 | -0.2436 | 0.1179 | 0.0388597 |
| rs112875651 | 8 | A | G | -0.07727 | 0.00418284 | 4.60E-78 | -0.2807 | 0.1196 | 0.0188999 |
| rs114863007 | 6 | A | G | -0.0480587 | 0.00692094 | 4.10E-13 | 0.0904 | 0.1648 | 0.5835 |
| rs115413710 | 6 | C | T | 0.0604013 | 0.0097982 | 8.10E-12 | 0.1917 | 0.2358 | 0.4163 |
| rs11789603 | 9 | T | C | 0.04771 | 0.00648318 | 7.60E-14 | 0.586 | 0.2213 | 0.00808295 |
| rs1260326 | 2 | C | T | -0.0762658 | 0.00411631 | 1.60E-77 | -0.193 | 0.1221 | 0.1138 |
| rs12740374 | 1 | T | G | -0.0589449 | 0.00484565 | 3.30E-34 | 0.0444 | 0.1415 | 0.7539 |
| rs13108218 | 4 | G | A | -0.0343116 | 0.00417075 | 1.60E-17 | 0.1615 | 0.1248 | 0.1955 |
| rs141469619 | 11 | G | A | 0.125151 | 0.0212786 | 1.30E-10 | -1.1058 | 0.9416 | 0.2402 |
| rs142158911 | 19 | A | G | -0.0922557 | 0.00631055 | 6.70E-50 | -0.0106 | 0.1915 | 0.9558 |
| rs1461729 | 8 | G | A | 0.0858304 | 0.0066974 | 1.30E-38 | -0.0483 | 0.1701 | 0.776499 |
| rs1800961 | 20 | T | C | -0.0717226 | 0.0117677 | 1.60E-09 | -0.1298 | 0.2776 | 0.6401 |
| rs183130 | 16 | T | C | 0.0559719 | 0.00430633 | 7.20E-41 | 0.2623 | 0.1295 | 0.0427602 |
| rs2229738 | 11 | T | C | -0.0410344 | 0.00808345 | 3.60E-08 | 0.2043 | 0.1634 | 0.2112 |
| rs2326077 | 8 | T | C | -0.0284526 | 0.00426919 | 1.60E-11 | 0.027 | 0.1193 | 0.8207 |
| rs261290 | 15 | C | T | -0.112315 | 0.00424505 | 1.20E-157 | -0.13 | 0.1192 | 0.2754 |
| rs2737245 | 8 | T | G | -0.0285112 | 0.00450206 | 2.00E-10 | -0.1001 | 0.1262 | 0.4275 |
| rs2740488 | 9 | C | A | -0.049166 | 0.004574 | 7.20E-27 | -0.0919 | 0.1462 | 0.5298 |
| rs28383314 | 6 | C | T | 0.0403992 | 0.00415433 | 1.70E-19 | 0.0817 | 0.1385 | 0.5553 |
| rs34955778 | 16 | C | T | -0.029365 | 0.00408638 | 1.30E-12 | -0.2522 | 0.1171 | 0.0312702 |
| rs3770586 | 2 | T | C | -0.0238966 | 0.00404591 | 1.00E-09 | -0.2756 | 0.1183 | 0.0198198 |
| rs3822855 | 6 | T | G | 0.0235759 | 0.00410926 | 6.40E-09 | -0.146 | 0.1171 | 0.2126 |
| rs3843482 | 5 | G | T | 0.0424902 | 0.00416334 | 2.70E-25 | -0.0359 | 0.1169 | 0.758901 |
| rs4299376 | 2 | T | G | -0.0315084 | 0.00431374 | 7.30E-14 | 0.0321 | 0.1412 | 0.82 |
| rs4561509 | 17 | A | G | 0.0239181 | 0.00404085 | 7.60E-10 | -0.1154 | 0.1163 | 0.321 |
| rs534417 | 1 | G | A | 0.0385998 | 0.00607001 | 1.10E-10 | -0.0456 | 0.1833 | 0.8037 |
| rs55747707 | 7 | A | G | -0.0611785 | 0.00501785 | 5.90E-34 | 0.23 | 0.151 | 0.1279 |
| rs56322906 | 19 | A | G | -0.104445 | 0.0109466 | 2.20E-21 | 0.3322 | 0.2373 | 0.1615 |
| rs58542926 | 19 | T | C | -0.152376 | 0.00767948 | 1.50E-91 | -0.1741 | 0.2367 | 0.462 |
| rs59774409 | 19 | T | C | 0.0427981 | 0.00727468 | 4.40E-09 | 0.2106 | 0.2833 | 0.4572 |
| rs633695 | 15 | G | A | 0.0838432 | 0.0044443 | 1.10E-79 | 0.0326 | 0.1256 | 0.795 |
| rs6547409 | 2 | T | C | -0.084643 | 0.00923521 | 4.30E-22 | 0.0269 | 0.2154 | 0.9007 |
| rs6602911 | 13 | T | C | 0.0279094 | 0.00419569 | 5.80E-11 | -0.1205 | 0.1217 | 0.322 |
| rs672889 | 2 | G | T | 0.0748648 | 0.00579704 | 7.30E-41 | -0.1428 | 0.1632 | 0.3814 |
| rs6882345 | 5 | A | G | 0.0456949 | 0.0041739 | 3.60E-29 | -0.0329 | 0.1232 | 0.7896 |
| rs6938647 | 6 | C | A | -0.0475077 | 0.00494185 | 3.30E-23 | -0.164 | 0.1486 | 0.2698 |
| rs72997616 | 11 | A | C | -0.0647308 | 0.00693225 | 8.40E-21 | -0.4042 | 0.1886 | 0.03209 |
| rs73013176 | 19 | C | T | -0.102018 | 0.0193941 | 2.90E-08 | 1.1864 | 0.8572 | 0.1663 |
| rs75406471 | 10 | A | G | -0.0352293 | 0.00558407 | 4.20E-10 | 0.1011 | 0.1665 | 0.5437 |
| rs7707394 | 5 | A | G | 0.0265334 | 0.0041973 | 1.50E-10 | -0.011 | 0.1197 | 0.9271 |
| rs77960347 | 18 | G | A | 0.276962 | 0.017596 | 5.70E-58 | -0.1125 | 0.6686 | 0.8664 |
| rs7970695 | 12 | A | G | -0.032523 | 0.00415946 | 2.00E-15 | -0.162 | 0.1185 | 0.1715 |
| rs870526 | 2 | T | C | -0.0321934 | 0.00403392 | 2.80E-16 | 0.1039 | 0.1189 | 0.3821 |
| rs9295128 | 6 | T | G | -0.203104 | 0.0159328 | 2.90E-39 | -0.412 | 0.5476 | 0.4519 |
| rs9304381 | 18 | T | C | 0.0738115 | 0.00523016 | 3.80E-46 | 0.0433 | 0.1522 | 0.776101 |

SNP: single nucleotide polymorphism; Chr: chromosome; EA: effect allele; OA: other allele; Beta was obtained by allele-related effects; SE: standard error; F, F-statistic; PUFA: Polyunsaturated fatty acids; VWD: Von Willebrand Disease

**3.Supplementary Table 3**IVs in MVMR analysis.

| **Exposure** | **SNP** | **EA** | **OA** | **Beta** | **SE** | **Pval** |
| --- | --- | --- | --- | --- | --- | --- |
| **Hypothyroidism** | rs1002687 | A | G | 0.0038 | 0.0106 | 0.7165 |
|  | rs10075764 | G | A | -0.057 | 0.0104 | 4.26E-08 |
|  | rs10096633 | T | C | -0.0455 | 0.0155 | 0.00342098 |
|  | rs10126000 | A | C | -0.0683 | 0.0104 | 5.13E-11 |
|  | rs102275 | C | T | -0.0368 | 0.0102 | 0.000325402 |
|  | rs1065386 | C | G | -0.1183 | 0.0125 | 3.93E-21 |
|  | rs1065853 | T | G | 0.0266 | 0.0231 | 0.2506 |
|  | rs1079418 | G | A | -0.0657 | 0.011 | 2.14E-09 |
|  | rs11171710 | A | G | -0.0698 | 0.01 | 3.19E-12 |
|  | rs112875651 | A | G | 0.0027 | 0.0103 | 0.7948 |
|  | rs115478735 | T | A | 0.019 | 0.0129 | 0.1399 |
|  | rs11675342 | T | C | 0.0906 | 0.01 | 1.40E-19 |
|  | rs11875260 | G | A | 0.0751 | 0.0135 | 2.54E-08 |
|  | rs12117927 | A | C | 0.0627 | 0.0105 | 2.29E-09 |
|  | rs12582330 | T | G | -0.061 | 0.0109 | 2.05E-08 |
|  | rs12593201 | A | G | 0.0905 | 0.0112 | 7.69E-16 |
|  | rs1260326 | C | T | -0.0018 | 0.0102 | 0.8564 |
|  | rs12740374 | T | G | 0.0135 | 0.0121 | 0.2648 |
|  | rs12984428 | A | G | -0.0659 | 0.0102 | 1.11E-10 |
|  | rs13090803 | T | G | 0.0829 | 0.0128 | 9.00E-11 |
|  | rs13108218 | G | A | -0.0097 | 0.0104 | 0.3506 |
|  | rs1364450 | C | A | 0.0886 | 0.0139 | 1.97E-10 |
|  | rs141469619 | G | A | 0.0701 | 0.06 | 0.2426 |
|  | rs142158911 | A | G | -0.0122 | 0.0162 | 0.450199 |
|  | rs142997491 | G | A | 0.2385 | 0.0412 | 7.02E-09 |
|  | rs1432806 | G | A | 0.0583 | 0.0105 | 2.89E-08 |
|  | rs1461729 | G | A | 0.0207 | 0.0155 | 0.1821 |
|  | rs1534430 | T | C | -0.086 | 0.0101 | 1.44E-17 |
|  | rs1800961 | T | C | 0.0054 | 0.0269 | 0.8407 |
|  | rs183130 | T | C | -0.0022 | 0.0108 | 0.8411 |
|  | rs187707293 | A | T | 0.2419 | 0.044 | 3.99E-08 |
|  | rs1883711 | C | G | -0.0084 | 0.0257 | 0.744501 |
|  | rs199717562 | G | A | -0.0176 | 0.0165 | 0.2858 |
|  | rs2111485 | G | A | 0.0813 | 0.0102 | 1.43E-15 |
|  | rs2114702 | A | T | 0.07 | 0.0111 | 3.00E-10 |
|  | rs2229738 | T | C | -8.00E-04 | 0.0175 | 0.965 |
|  | rs2234167 | A | G | 0.0825 | 0.015 | 3.75E-08 |
|  | rs229528 | T | C | 0.0903 | 0.01 | 2.31E-19 |
|  | rs2326077 | T | C | -0.0235 | 0.0104 | 0.02467 |
|  | rs2378390 | A | G | 0.0141 | 0.0182 | 0.4389 |
|  | rs2412976 | G | C | 0.0637 | 0.0103 | 5.47E-10 |
|  | rs2445608 | A | G | -0.0593 | 0.0101 | 3.79E-09 |
|  | rs244685 | G | T | -0.0858 | 0.0132 | 7.06E-11 |
|  | rs261290 | C | T | -0.0155 | 0.0103 | 0.1325 |
|  | rs2737245 | T | G | -0.0028 | 0.0109 | 0.7977 |
|  | rs28418426 | C | T | 0.1877 | 0.0133 | 2.21E-45 |
|  | rs2988277 | T | C | 0.0593 | 0.0106 | 2.49E-08 |
|  | rs307558 | A | G | -0.0688 | 0.0119 | 8.01E-09 |
|  | rs3087243 | A | G | -0.1466 | 0.0102 | 4.77E-47 |
|  | rs3118469 | T | A | 0.0803 | 0.0106 | 3.82E-14 |
|  | rs3184504 | C | T | -0.1734 | 0.0102 | 7.50E-65 |
|  | rs34955778 | C | T | 0.0147 | 0.01 | 0.1403 |
|  | rs3775291 | T | C | -0.0649 | 0.0108 | 1.65E-09 |
|  | rs3822855 | T | G | -0.0026 | 0.0101 | 0.7944 |
|  | rs3843482 | G | T | -0.0147 | 0.0101 | 0.1437 |
|  | rs4299376 | T | G | 0.0135 | 0.0113 | 0.2314 |
|  | rs434294 | G | A | -0.0683 | 0.0109 | 3.36E-10 |
|  | rs4409785 | C | T | 0.1069 | 0.0133 | 8.04E-16 |
|  | rs4529854 | T | C | -0.0768 | 0.0107 | 6.56E-13 |
|  | rs4835534 | C | T | -0.1421 | 0.0132 | 7.06E-27 |
|  | rs4860948 | A | T | -0.0185 | 0.0117 | 0.1129 |
|  | rs4986970 | T | A | 0.0258 | 0.0283 | 0.362 |
|  | rs534417 | G | A | 0.0336 | 0.0147 | 0.0223702 |
|  | rs55747707 | A | G | 0.0027 | 0.0126 | 0.8308 |
|  | rs56322906 | A | G | 0.0403 | 0.0222 | 0.0692197 |
|  | rs5754102 | A | C | 0.0415 | 0.0164 | 0.0115601 |
|  | rs58542926 | T | C | 0.0124 | 0.0191 | 0.515101 |
|  | rs61759532 | T | C | 0.0905 | 0.0122 | 1.42E-13 |
|  | rs61877856 | T | C | -0.0658 | 0.0115 | 1.14E-08 |
|  | rs633695 | G | A | 0.0067 | 0.0108 | 0.5371 |
|  | rs6547409 | T | C | -0.0023 | 0.0215 | 0.9143 |
|  | rs6602911 | T | C | -0.0044 | 0.0103 | 0.6704 |
|  | rs6679677 | A | C | 0.3637 | 0.0159 | 2.39E-115 |
|  | rs672889 | G | T | -0.0131 | 0.0146 | 0.3702 |
|  | rs6882345 | A | G | -0.0048 | 0.0104 | 0.6445 |
|  | rs6908626 | T | G | 0.1441 | 0.0141 | 2.04E-24 |
|  | rs6938647 | C | A | 0.0048 | 0.0126 | 0.7052 |
|  | rs7030280 | T | C | 0.2075 | 0.0108 | 1.02E-82 |
|  | rs71508903 | T | C | 0.0934 | 0.0125 | 9.34E-14 |
|  | rs7223956 | C | T | -0.0902 | 0.0144 | 4.27E-10 |
|  | rs72997616 | A | C | -0.0066 | 0.0161 | 0.680801 |
|  | rs73013176 | C | T | -0.0592 | 0.0541 | 0.2739 |
|  | rs73192661 | T | C | -0.1061 | 0.01 | 4.05E-26 |
|  | rs736374 | A | G | 0.0832 | 0.0103 | 6.00E-16 |
|  | rs7441808 | G | A | 0.0766 | 0.0111 | 5.17E-12 |
|  | rs7574865 | G | T | -0.1321 | 0.0117 | 1.67E-29 |
|  | rs7742626 | C | T | 0.0686 | 0.0116 | 3.41E-09 |
|  | rs77960347 | G | A | -0.0341 | 0.0478 | 0.4758 |
|  | rs7831074 | G | C | -0.0297 | 0.0157 | 0.0584494 |
|  | rs79490353 | C | T | 0.2006 | 0.0349 | 8.82E-09 |
|  | rs7970695 | A | G | 0.021 | 0.0101 | 0.0382798 |
|  | rs7990020 | C | A | 0.0577 | 0.0101 | 9.97E-09 |
|  | rs881858 | A | G | 0.0665 | 0.0108 | 8.46E-10 |
|  | rs911760 | A | C | 0.0879 | 0.0125 | 1.95E-12 |
|  | rs926103 | C | T | -0.0678 | 0.0104 | 7.65E-11 |
|  | rs9271365 | G | T | 0.2484 | 0.0105 | 4.91E-123 |
|  | rs9277559 | C | T | -0.133 | 0.012 | 1.86E-28 |
|  | rs9295128 | T | G | -0.0144 | 0.0422 | 0.7326 |
|  | rs9304381 | T | C | 0.0082 | 0.0128 | 0.522701 |
|  | rs9497965 | T | C | 0.0827 | 0.0102 | 3.71E-16 |
|  | rs9511151 | A | G | -0.0976 | 0.0106 | 3.08E-20 |
|  | rs9616847 | T | A | 0.0273 | 0.0101 | 0.007006 |
|  | rs964184 | C | G | -0.0142 | 0.0141 | 0.3151 |
|  | rs9902341 | T | C | 0.0801 | 0.0129 | 4.68E-10 |
| **Polyunsaturated fatty acids** | rs1002687 | A | G | 0.0959946 | 0.00420718 | 2.00E-120 |
|  | rs10075764 | G | A | 0.00695811 | 0.00426399 | 0.11 |
|  | rs10096633 | T | C | -0.0339303 | 0.00610477 | 3.10E-08 |
|  | rs10126000 | A | C | -0.00201711 | 0.00418259 | 0.75 |
|  | rs102275 | C | T | -0.0974089 | 0.00421144 | 3.20E-125 |
|  | rs1065386 | C | G | 0.0126 | 0.00417201 | 0.01 |
|  | rs1065853 | T | G | -0.169688 | 0.00742914 | 3.70E-118 |
|  | rs1079418 | G | A | -0.00301504 | 0.00437443 | 0.4 |
|  | rs11171710 | A | G | 0.000309704 | 0.00407856 | 0.97 |
|  | rs112875651 | A | G | -0.07727 | 0.00418284 | 4.60E-78 |
|  | rs115478735 | T | A | 0.0373598 | 0.00521074 | 7.40E-14 |
|  | rs11675342 | T | C | -0.00572105 | 0.00407795 | 0.15 |
|  | rs11875260 | G | A | 0.000465877 | 0.0057013 | 0.98 |
|  | rs12117927 | A | C | 0.00251129 | 0.00414607 | 0.55 |
|  | rs12582330 | T | G | -0.000105878 | 0.00454087 | 0.84 |
|  | rs12593201 | A | G | -0.00724742 | 0.00465493 | 0.0759994 |
|  | rs1260326 | C | T | -0.0762658 | 0.00411631 | 1.60E-77 |
|  | rs12740374 | T | G | -0.0589449 | 0.00484565 | 3.30E-34 |
|  | rs12984428 | A | G | 0.000521514 | 0.0040954 | 0.67 |
|  | rs13090803 | T | G | 0.00142141 | 0.00495501 | 0.86 |
|  | rs13108218 | G | A | -0.0343116 | 0.00417075 | 1.60E-17 |
|  | rs1364450 | C | A | -0.0126977 | 0.00573357 | 0.0290001 |
|  | rs141469619 | G | A | 0.125151 | 0.0212786 | 1.30E-10 |
|  | rs142158911 | A | G | -0.0922557 | 0.00631055 | 6.70E-50 |
|  | rs142997491 | G | A | 0.00593661 | 0.0183469 | 0.74 |
|  | rs1432806 | G | A | -0.00551649 | 0.00424162 | 0.14 |
|  | rs1461729 | G | A | 0.0858304 | 0.0066974 | 1.30E-38 |
|  | rs1534430 | T | C | -0.000811021 | 0.0041356 | 0.98 |
|  | rs1800961 | T | C | -0.0717226 | 0.0117677 | 1.60E-09 |
|  | rs183130 | T | C | 0.0559719 | 0.00430633 | 7.20E-41 |
|  | rs187707293 | A | T | 0.0132936 | 0.0197632 | 0.53 |
|  | rs1883711 | C | G | 0.09328 | 0.0118393 | 9.50E-17 |
|  | rs199717562 | G | A | -0.0356887 | 0.00541346 | 1.10E-11 |
|  | rs2111485 | G | A | -0.0100176 | 0.00411205 | 0.0109999 |
|  | rs2114702 | A | T | -0.00325276 | 0.00435836 | 0.4 |
|  | rs2229738 | T | C | -0.0410344 | 0.00808345 | 3.60E-08 |
|  | rs2234167 | A | G | -0.00303758 | 0.00590488 | 0.52 |
|  | rs229528 | T | C | -0.00159298 | 0.00408803 | 0.85 |
|  | rs2326077 | T | C | -0.0284526 | 0.00426919 | 1.60E-11 |
|  | rs2378390 | A | G | -0.0324241 | 0.00581303 | 1.20E-08 |
|  | rs2412976 | G | C | -0.0101427 | 0.00412177 | 0.0269998 |
|  | rs2445608 | A | G | -0.00166804 | 0.00409563 | 0.77 |
|  | rs244685 | G | T | -0.0102009 | 0.0060841 | 0.0619998 |
|  | rs261290 | C | T | -0.112315 | 0.00424505 | 1.20E-157 |
|  | rs2737245 | T | G | -0.0285112 | 0.00450206 | 2.00E-10 |
|  | rs28418426 | C | T | 0.00587893 | 0.00442464 | 0.17 |
|  | rs2988277 | T | C | -0.0077087 | 0.00412213 | 0.0810009 |
|  | rs307558 | A | G | 0.00326852 | 0.00500959 | 0.64 |
|  | rs3087243 | A | G | 0.00546309 | 0.00403911 | 0.0649995 |
|  | rs3118469 | T | A | -0.000198628 | 0.00439838 | 0.93 |
|  | rs3184504 | C | T | 0.0267131 | 0.00403671 | 1.30E-11 |
|  | rs34955778 | C | T | -0.029365 | 0.00408638 | 1.30E-12 |
|  | rs3775291 | T | C | 0.00510647 | 0.00440511 | 0.23 |
|  | rs3822855 | T | G | 0.0235759 | 0.00410926 | 6.40E-09 |
|  | rs3843482 | G | T | 0.0424902 | 0.00416334 | 2.70E-25 |
|  | rs4299376 | T | G | -0.0315084 | 0.00431374 | 7.30E-14 |
|  | rs434294 | G | A | -0.00574717 | 0.00431702 | 0.12 |
|  | rs4409785 | C | T | -0.00754734 | 0.00531918 | 0.0980009 |
|  | rs4529854 | T | C | 0.000797007 | 0.00422097 | 0.69 |
|  | rs4835534 | C | T | -0.0106287 | 0.00493502 | 0.0460002 |
|  | rs4860948 | A | T | 0.0337639 | 0.00470169 | 4.90E-13 |
|  | rs4986970 | T | A | -0.0638502 | 0.011169 | 1.10E-08 |
|  | rs534417 | G | A | 0.0385998 | 0.00607001 | 1.10E-10 |
|  | rs55747707 | A | G | -0.0611785 | 0.00501785 | 5.90E-34 |
|  | rs56322906 | A | G | -0.104445 | 0.0109466 | 2.20E-21 |
|  | rs5754102 | A | C | -0.0288301 | 0.00526871 | 2.30E-08 |
|  | rs58542926 | T | C | -0.152376 | 0.00767948 | 1.50E-91 |
|  | rs61759532 | T | C | -0.00620282 | 0.00481279 | 0.3 |
|  | rs61877856 | T | C | 0.000714657 | 0.00448586 | 0.91 |
|  | rs633695 | G | A | 0.0838432 | 0.0044443 | 1.10E-79 |
|  | rs6547409 | T | C | -0.084643 | 0.00923521 | 4.30E-22 |
|  | rs6602911 | T | C | 0.0279094 | 0.00419569 | 5.80E-11 |
|  | rs6679677 | A | C | -0.0135286 | 0.00669344 | 0.0659994 |
|  | rs672889 | G | T | 0.0748648 | 0.00579704 | 7.30E-41 |
|  | rs6882345 | A | G | 0.0456949 | 0.0041739 | 3.60E-29 |
|  | rs6908626 | T | G | -0.00551802 | 0.00530039 | 0.32 |
|  | rs6938647 | C | A | -0.0475077 | 0.00494185 | 3.30E-23 |
|  | rs7030280 | T | C | 0.011697 | 0.00428808 | 0.00409996 |
|  | rs71508903 | T | C | 0.000527308 | 0.00515007 | 0.87 |
|  | rs7223956 | C | T | 0.00555555 | 0.00576677 | 0.27 |
|  | rs72997616 | A | C | -0.0647308 | 0.00693225 | 8.40E-21 |
|  | rs73013176 | C | T | -0.102018 | 0.0193941 | 2.90E-08 |
|  | rs73192661 | T | C | 0.00507135 | 0.00407147 | 0.13 |
|  | rs736374 | A | G | 0.00326369 | 0.00420343 | 0.630001 |
|  | rs7441808 | G | A | 0.00402573 | 0.00439919 | 0.3 |
|  | rs7574865 | G | T | -0.000127158 | 0.00482959 | 0.92 |
|  | rs7742626 | C | T | 0.00574118 | 0.00448744 | 0.13 |
|  | rs77960347 | G | A | 0.276962 | 0.017596 | 5.70E-58 |
|  | rs7831074 | G | C | 0.0291217 | 0.00504193 | 9.50E-09 |
|  | rs79490353 | C | T | -0.0500964 | 0.0128967 | 0.000179999 |
|  | rs7970695 | A | G | -0.032523 | 0.00415946 | 2.00E-15 |
|  | rs7990020 | C | A | -0.00916363 | 0.00404932 | 0.0539995 |
|  | rs881858 | A | G | 0.00585307 | 0.0043651 | 0.12 |
|  | rs911760 | A | C | -0.0104188 | 0.00522985 | 0.0350002 |
|  | rs926103 | C | T | -0.00110445 | 0.00425919 | 0.85 |
|  | rs9271365 | G | T | -0.0243384 | 0.00406417 | 8.70E-07 |
|  | rs9277559 | C | T | 0.0107444 | 0.00487183 | 0.021 |
|  | rs9295128 | T | G | -0.203104 | 0.0159328 | 2.90E-39 |
|  | rs9304381 | T | C | 0.0738115 | 0.00523016 | 3.80E-46 |
|  | rs9497965 | T | C | 0.00179297 | 0.00410938 | 0.760001 |
|  | rs9511151 | A | G | -0.00790341 | 0.00424895 | 0.0580003 |
|  | rs9616847 | T | A | 0.0234318 | 0.00415401 | 3.50E-08 |
|  | rs964184 | C | G | -0.15015 | 0.00593524 | 5.20E-146 |
|  | rs9902341 | T | C | -0.00581001 | 0.00542942 | 0.39 |

SNP: single nucleotide polymorphism; Chr: chromosome; EA: effect allele; OA: other allele; Beta was obtained by allele-related effects; SE: standard error; PUFA: Polyunsaturated fatty acids
